# Supplementary material for: Neural correlates of cognitive ability and visuo-motor speed: Validation of IDoCT on UK Biobank Data
Source: Imaging Neurosci (Camb). 2024 Feb 9;2:imag-2-00087. doi: 10.1162/imag_a_00087 (PMC12224415; doi:10.1162/imag_a_00087)
Supplement: Supplementary Material [file imag_a_00087-supp.pdf]

APPENDIX A

Table A1 List of structural MRI (sMRI) imaging features. The imaging data consisted of 26 measures of fractional anisotropy (FA) from diffusion weighted images (DWI), and 139 volume, 43 intensity and 60 cortical thickness measures from T1-weighted structural magnetic resonance images (MRI). The imaging features included in the study are provided in the Table.

Dataset	Features
**FA**	• Tract anterior thalamic radiation (right)<br>• Tract cingulate gyrus part of cingulum (right)<br>• Tract corticospinal tract (right)<br>• Tract inferior longitudinal fasciculus (right)<br>• Tract medial lemniscus (right)<br>• Tract posterior thalamic radiation (right)<br>• Tract superior thalamic radiation (right)<br>• Tract anterior thalamic radiation (left)<br>• Tract cingulate gyrus part of cingulum (left)<br>• Tract corticospinal tract (left)<br>• Tract inferior longitudinal fasciculus (left)<br>• Tract posterior thalamic radiation (left)<br>• Tract superior longitudinal fasciculus (left)<br>• Tract uncinate fasciculus (left)<br>• Tract acoustic radiation (left)<br>• Tract parahippocampal part of cingulum (left)<br>• Tract forceps major<br>• Tract inferior fronto occipital fasciculus (left)<br>• Tract medial lemniscus (left)<br>• Tract superior thalamic radiation (left)<br>• Tract acoustic radiation (right)<br>• Tract parahippocampal part of cingulum (right)<br>• Tract forceps minor<br>• Tract inferior fronto occipital fasciculus (right)<br>• Tract middle cerebellar peduncle<br>• Tract superior longitudinal fasciculus (right)
**Thickness**	• Cuneus (left hemisphere)<br>• Lateraloccipital (left hemisphere)<br>• Lingual (left hemisphere)<br>• Parsorbitalis (left hemisphere)<br>• Posteriorcingulate (left hemisphere)<br>• Precuneus (left hemisphere)<br>• Superiorparietal (left hemisphere)<br>• Entorhinal (right hemisphere)<br>• Isthmuscingulate (right hemisphere)<br>• Lateralorbitofrontal (right hemisphere)<br>• Parsopercularis (right hemisphere)<br>• Parstriangularis (right hemisphere)<br>• Precentral (right hemisphere)<br>• Superiortemporal (right hemisphere)<br>• Entorhinal (left hemisphere)<br>• Lateralorbitofrontal (left hemisphere)<br>• Medialorbitofrontal (left hemisphere)<br>• Parstriangularis (left hemisphere)<br>• Precentral (left hemisphere)<br>• Rostralanteriorcingulate (left hemisphere)<br>• Superiortemporal (left hemisphere)<br>• Fusiform (right hemisphere)<br>• Lateraloccipital (right hemisphere)<br>• Lingual (right hemisphere)<br>• Parsorbitalis (right hemisphere)<br>• Pericalcarine (right hemisphere)<br>• Precuneus (right hemisphere)

	• Supramarginal (right hemisphere)<br>• Caudalmiddlefrontal (left hemisphere)<br>• Inferiorparietal (left hemisphere)<br>• Isthmuscingulate (left hemisphere)<br>• Parahippocampal (left hemisphere)<br>• Parsopercularis (left hemisphere)<br>• Postcentral (left hemisphere)<br>• Superiorfrontal (left hemisphere)<br>• Transversetemporal (left hemisphere)<br>• Caudalanteriorcingulate (right hemisphere)<br>• Cuneus (right hemisphere)<br>• Inferiortemporal (right hemisphere)<br>• Middletemporal (right hemisphere)<br>• Paracentral (right hemisphere)<br>• Posteriorcingulate (right hemisphere)<br>• Rostralmiddlefrontal (right hemisphere)<br>• Superiorparietal (right hemisphere)<br>• Transversetemporal (right hemisphere)<br>• Caudalanteriorcingulate (left hemisphere)<br>• Fusiform (left hemisphere)<br>• Inferiortemporal (left hemisphere)<br>• Middletemporal (left hemisphere)<br>• Paracentral (left hemisphere)<br>• Pericalcarine (left hemisphere)<br>• Rostralmiddlefrontal (left hemisphere)<br>• Supramarginal (left hemisphere)<br>• Caudalmiddlefrontal (right hemisphere)<br>• Inferiorparietal (right hemisphere)<br>• Medialorbitofrontal (right hemisphere)<br>• Parahippocampal (right hemisphere)<br>• Postcentral (right hemisphere)<br>• Rostralanteriorcingulate (right hemisphere)<br>• Superiorfrontal (right hemisphere)
**Volume**	• Insular Cortex (left)<br>• Inferior Frontal Gyrus pars triangularis (left)<br>• Precentral Gyrus (left)<br>• Temporal Pole (left)<br>• Superior Temporal Gyrus posterior division (left)<br>• Middle Temporal Gyrus temporooccipital part (left)<br>• Inferior Temporal Gyrus anterior division (left)<br>• Postcentral Gyrus (left)<br>• Supramarginal Gyrus anterior division (left)<br>• Supramarginal Gyrus posterior division (left)<br>• Lateral Occipital Cortex superior division (right)<br>• Frontal Medial Cortex (right)<br>• Subcallosal Cortex (right)<br>• Paracingulate Gyrus (right)<br>• Precuneous Cortex (right)<br>• Cuneal Cortex (right)<br>• Parahippocampal Gyrus anterior division (right)<br>• Temporal Fusiform Cortex anterior division (right)<br>• Temporal Fusiform Cortex posterior division (left)<br>• Frontal Operculum Cortex (left)<br>• Parietal Operculum Cortex (left)<br>• Planum Polare (left)<br>• Supracalcarine Cortex (left)<br>• Occipital Pole (left)<br>• Caudate (left)<br>• Hippocampus (left)<br>• Ventral Striatum (right)<br>• I IV Cerebellum (left)

<table><tr><td></td><td>

- VI Cerebellum (left)
- Crus II Cerebellum (left)
- Crus II Cerebellum (right)
- VIIIa Cerebellum (right)
- IX Cerebellum (left)
- IX Cerebellum (right)
- Insular Cortex (right)
- Inferior Frontal Gyrus pars triangularis (right)
- Precentral Gyrus (right)
- Temporal Pole (right)
- Superior Temporal Gyrus posterior division (right)
- Middle Temporal Gyrus temporooccipital part (right)
- Inferior Temporal Gyrus anterior division (right)
- Postcentral Gyrus (right)
- Supramarginal Gyrus anterior division (right)
- Supramarginal Gyrus posterior division (right)
- Lateral Occipital Cortex superior division (left)
- Frontal Medial Cortex (left)
- Subcallosal Cortex (left)
- Paracingulate Gyrus (left)
- Precuneous Cortex (left)
- Cuneal Cortex (left)
- Parahippocampal Gyrus anterior division (left)
- Temporal Fusiform Cortex anterior division (left)
- Temporal Fusiform Cortex posterior division (right)
- Frontal Operculum Cortex (right)
- Parietal Operculum Cortex (right)
- Planum Polare (right)
- Supracalcarine Cortex (right)
- Occipital Pole (right)
- Caudate (right)
- Hippocampus (right)
- Ventral Striatum (left)
- Brain Stem
- V Cerebellum (right)
- Crus I Cerebellum (right)
- Crus II Cerebellum (vermis)
- VIIIa Cerebellum (vermis)
- VIIIb Cerebellum (right)
- IX Cerebellum (vermis)
- X Cerebellum (right)
- Frontal Pole (right)
- Superior Frontal Gyrus (right)
- Middle Frontal Gyrus (right)
- Inferior Frontal Gyrus pars opercularis (right)
- Superior Temporal Gyrus anterior division (right)
- Middle Temporal Gyrus anterior division (right)
- Middle Temporal Gyrus posterior division (right)
- Inferior Temporal Gyrus posterior division (right)
- Inferior Temporal Gyrus temporooccipital part (right)
- Superior Parietal Lobule (right)
- Angular Gyrus (right)
- Lateral Occipital Cortex inferior division (left)
- Intracalcarine Cortex (left)
- Juxtapositional Lobule Cortex formerly Supplementary Motor Cortex (left)
- Cingulate Gyrus anterior division (left)
- Cingulate Gyrus posterior division (left)
- Frontal Orbital Cortex (left)
- Parahippocampal Gyrus posterior division (left)
- Lingual Gyrus (left)
- Temporal Occipital Fusiform Cortex (right)

</td></tr></table>

	• Occipital Fusiform Gyrus (right)<br>• Central Opercular Cortex (right)<br>• Heschl s Gyrus includes H1 and H2 (right)<br>• Planum Temporale (right)<br>• Thalamus (right)<br>• Putamen (right)<br>• Pallidum (right)<br>• Amygdala (left)<br>• I IV Cerebellum (right)<br>• VI Cerebellum (vermis)<br>• Crus I Cerebellum (left)<br>• VIIb Cerebellum (left)<br>• VIIb Cerebellum (right)<br>• VIIIb Cerebellum (left)<br>• X Cerebellum (left)<br>• Frontal Pole (left)<br>• Superior Frontal Gyrus (left)<br>• Middle Frontal Gyrus (left)<br>• Inferior Frontal Gyrus pars opercularis (left)<br>• Superior Temporal Gyrus anterior division (left)<br>• Middle Temporal Gyrus anterior division (left)<br>• Middle Temporal Gyrus posterior division (left)<br>• Inferior Temporal Gyrus posterior division (left)<br>• Inferior Temporal Gyrus temporooccipital part (left)<br>• Superior Parietal Lobule (left)<br>• Angular Gyrus (left)<br>• Lateral Occipital Cortex inferior division (right)<br>• Intracalcarine Cortex (right)<br>• Juxtapositional Lobule Cortex formerly Supplementary Motor Cortex (right)<br>• Cingulate Gyrus anterior division (right)<br>• Cingulate Gyrus posterior division (right)<br>• Frontal Orbital Cortex (right)<br>• Parahippocampal Gyrus posterior division (right)<br>• Lingual Gyrus (right)<br>• Temporal Occipital Fusiform Cortex (left)<br>• Occipital Fusiform Gyrus (left)<br>• Central Opercular Cortex (left)<br>• Heschl s Gyrus includes H1 and H2 (left)<br>• Planum Temporale (left)<br>• Thalamus (left)<br>• Putamen (left)<br>• Pallidum (left)<br>• Amygdala (right)<br>• V Cerebellum (left)<br>• VI Cerebellum (right)<br>• Crus I Cerebellum (vermis)<br>• VIIb Cerebellum (vermis)<br>• VIIIa Cerebellum (left)<br>• VIIIb Cerebellum (vermis)<br>• X Cerebellum (vermis)
**Intensity**	• Brain Stem (whole brain)<br>• CC Mid Posterior (whole brain)<br>• CC Mid Anterior (whole brain)<br>• Inf Lat Vent (left hemisphere)<br>• Cerebellum Cortex (left hemisphere)<br>• Amygdala (left hemisphere)<br>• VentralDC (left hemisphere)<br>• Lateral Ventricle (right hemisphere)<br>• Putamen (right hemisphere)<br>• Hippocampus (right hemisphere)<br>• 3rd Ventricle (whole brain)

	• CSF (whole brain)<br>• CC Central (whole brain)<br>• CC Anterior (whole brain)<br>• Lateral Ventricle (left hemisphere)<br>• Cerebellum White Matter (left hemisphere)<br>• Hippocampus (left hemisphere)<br>• Accumbens area (left hemisphere)<br>• Caudate (right hemisphere)<br>• Pallidum (right hemisphere)<br>• Choroid plexus (right hemisphere)<br>• 5th Ventricle (whole brain)<br>• Non WM hypointensities (whole brain)<br>• CC Posterior (whole brain)<br>• Thalamus Proper (left hemisphere)<br>• Putamen (left hemisphere)<br>• Vessel (left hemisphere)<br>• Inf Lat Vent (right hemisphere)<br>• Cerebellum Cortex (right hemisphere)<br>• Amygdala (right hemisphere)<br>• VentralDC (right hemisphere)<br>• 4th Ventricle (whole brain)<br>• WM hypointensities (whole brain)<br>• Optic Chiasm (whole brain)<br>• Caudate (left hemisphere)<br>• Pallidum (left hemisphere)<br>• Choroid plexus (left hemisphere)<br>• Cerebellum White Matter (right hemisphere)<br>• Thalamus Proper (right hemisphere)<br>• Accumbens area (right hemisphere)<br>• Vessel (right hemisphere)

Table A2 PubMed Central search criteria. Different search criteria were used to identify previous literature associated to the brain labels of the neural correlates of AS an DT. The list of the search criteria, together with the number of the papers that matched those criteria, is provided in the Table.

Brain region	PubMed search criteria	Number of papers
Hippocampus	(hippocampus[Title]) AND function[Title]	116
Superior temporal gyrus	((superior temporal gyrus[Title]) AND function)	85
Transverse temporal gyrus	(transverse temporal gyrus[Title]) OR ((Heschl's gyrus[Title]) AND function)	25
Medial Lemniscus	((((medial lemniscus[Title]) OR Reil's ribbon[Title]) OR Reil's band[Title])) OR medial lemniscus[Abstract]	99
Anterior corpus callosum	((corpus callosum[Title]) AND anterior[Abstract]) AND cognitive function	114
Medial Frontal Cortex	medial frontal cortex[Title]	102
Uncinate fasciculus	((uncinate fasciculus[Abstract]) AND uncinate fasciculus[Title]) AND function	47
Nucleus Accumbens	(accumbens[Title]) AND cognition[Body - Key Terms]	89
Caudal middle frontal gyrus	(((caudal middle frontal[Title]) OR caudal middle frontal[Abstract])) AND function	65
Inferior temporal gyrus	inferior temporal gyrus[Title]	12
Superior frontal gyrus	((superior frontal gyrus[Title]) AND function)	22
Parahippocampal part of cingulum	(((parahippocampal cingulum[Abstract]) OR parahippocampal cingulum[Title])) AND function	38
Forceps major	(((forceps major[Abstract]) OR forceps major[Title])) AND forceps major[Body - Key Terms]	92
Posterior thalamic radiations	(((posterior thalamic radiation[Abstract]) OR posterior thalamic radiation[Title])) AND function	105
Middle temporal gyrus	middle temporal area[Title]	31
Lateral orbitofrontal cortex	lateral orbitofrontal cortex[Title]	37
Occipital fusiform gyrus	(occipital fusiform gyrus[Abstract]) OR occipital fusiform gyrus[Title]	19
Lateral occipital cortex	(lateral occipital cortex[Title]) AND function	19
Amygdala	(amygdala[Title]) AND function[Title]	85
Frontal pole/lobe	((frontal lobe[Title]) AND cognition[Body - Key Terms])	97
Insular cortex	(((insular cortex[Title]))) AND cognition[Body - All Words]	62
Cerebellum	((cerebellum[Title])) AND cognitive function[Body - Key Terms]	117
Temporal fusiform cortex	(((temporal fusiform cortex[Title]) AND cognition[Body - Key Terms])) OR temporal fusiform cortex[Abstract]	12
Intra calcarine cortex	((intra-calcarine[Abstract]) OR intracalcarine[Abstract]) AND function[Body - All Words]	19
Occipital pole/lobe	occipital lobe[Title]	93

Table A3 IDoCT results of D and DS. List of words used in the PVT Task and the measures of D and DS derived from IDoCT.

Meaning	D	DS	Meaning	D	DS	Meaning	D	DS	Meaning	D	DS
crayon	0,52	0,52	confine	0,57	0,74	shore	0,51	0,7	adhere	0,67	0,69
nap	0,52	0,52	corroded	0,53	0,72	shovel	0,55	0,74	amiable	0,59	0,61
paper	0,6	0,61	equation	0,54	0,73	tent	0,59	0,6	arid	0,6	0,65
party	0,61	0,71	erode	0,59	0,65	belt	0,6	0,65	bestow	0,67	0,68
sick	0,59	0,61	fabricate	0,58	0,51	braid	0,57	0,75	cascade	0,54	0,71
big	0,58	0,63	frothy	0,58	0,61	broom	0,53	0,74	chaff	0,62	0,65
bus	0,53	0,71	gavel	0,62	0,76	brush	0,52	0,73	concave	0,68	0,66
egg	0,65	0,63	journal	0,62	0,82	buy	0,54	0,73	disarray	0,56	0,75
fun	0,61	0,62	knead	0,54	0,66	cactus	0,53	0,74	excavate	0,56	0,69
orange	0,66	0,73	ladle	0,52	0,67	hive	0,52	0,74	irate	0,54	0,64
tree	0,62	0,79	manger	0,76	0,75	lighthouse	0,51	0,69	jocular	0,56	0,68
triangle	0,53	0,72	masonry	0,59	0,64	mask	0,7	0,67	lustre	0,64	0,66
tricycle	0,52	0,71	molten	0,54	0,73	melted	0,56	0,77	offspring	0,57	0,77
under	0,54	0,69	odometer	0,73	0,74	railroad	0,57	0,77	ravine	0,65	0,69
zoo	0,56	0,7	oxidized	0,59	0,64	rubbish	0,59	0,63	receptacle	0,63	0,67
fireman	0,63	0,71	quibble	0,57	0,71	blanket	0,53	0,73	refurbish	0,55	0,61
jar	0,52	0,74	rural	0,61	0,65	bucket	0,56	0,71	refuse	0,68	0,64
log	0,51	0,72	satchel	0,52	0,74	calm	0,52	0,72	summit	0,58	0,6
pan	0,51	0,73	scholar	0,61	0,73	carry	0,53	0,73	abode	0,59	0,65
queen	0,52	0,75	sentry	0,57	0,7	castle	0,52	0,75	cubicle	0,63	0,76
run	0,58	0,59	shimmer	0,58	0,74	cattle	0,55	0,74	decrepit	0,54	0,73
ride	0,69	0,77	tarpaulin	0,55	0,62	fancy	0,63	0,77	effervescent	0,62	0,63
rocket	0,51	0,73	trench	0,52	0,74	metal	0,51	0,73	fiery	0,61	0,65
stick	0,64	0,63	unstable	0,54	0,72	net	0,52	0,73	figurine	0,53	0,73
tractor	0,53	0,74	attire	0,6	0,65	plastic	0,52	0,65	gregarious	0,57	0,62
wet	0,64	0,66	barren	0,59	0,7	porch	0,52	0,72	hydroponics	0,69	0,68
airplane	0,53	0,66	beacon	0,59	0,62	port	0,51	0,71	jovial	0,55	0,63
angry	0,65	0,56	broth	0,64	0,84	acrobat	0,53	0,72	labryinth	0,63	0,76
back (of)	0,54	0,73	camouflaged	0,55	0,78	cottage	0,51	0,69	noxious	0,56	0,74
beside	0,58	0,79	comrade	0,73	0,74	diamond	0,61	0,8	pachyderm	0,87	0,77
chair	0,68	0,75	frigid	0,67	0,75	envelope	0,51	0,72	panorama	0,52	0,65
coat	0,58	0,63	knick-knack	0,62	0,78	fellowship	0,52	0,67	quills	0,52	0,69
doctor	0,56	0,73	pageantry	0,69	0,76	gold	0,52	0,73	resplendent	0,58	0,63
fly	0,57	0,59	tarnished	0,62	0,73	instrument	0,52	0,74	silhouette	0,63	0,68
leaf	0,69	0,76	tattered	0,56	0,75	jewelry	0,51	0,72	slat	0,57	0,64
ocean	0,57	0,66	thicket	0,61	0,65	monument	0,57	0,71	striated	0,7	0,7
open	0,54	0,75	transact	0,56	0,7	pyramid	0,51	0,69	vortex	0,55	0,75
palace	0,52	0,73	turbulent	0,59	0,67	rose	0,54	0,74	celestial	0,64	0,66
riding	0,52	0,74	tusk	0,51	0,73	tortoise	0,51	0,69	cosmopolitan	0,63	0,66
voyage	0,52	0,74	depot	0,55	0,74	construct	0,54	0,76	trivet	0,76	0,87
windmill	0,52	0,75	distinguished	0,6	0,7	creek	0,62	0,83	verdant	0,65	0,68
garden	0,52	0,71	flamboyant	0,57	0,72	ferocious	0,52	0,7	affable	0,62	0,69
boulder	0,55	0,74	foal	0,56	0,62	flimsy	0,54	0,72	cherubic	0,63	0,64

**bouquet**	0,51	0,68	**lounging**	0,53	0,69	**hedge**	0,53	0,74	**coalesce**	0,74	0,73
**cable**	0,53	0,69	**tethered**	0,55	0,73	**incinerate**	0,58	0,74	**deluge**	0,59	0,63
**exhibit**	0,6	0,78	**zenith**	0,74	0,73	**instruct**	0,53	0,75	**didactic**	0,73	0,84
**haul**	0,59	0,69	**baubles**	0,58	0,73	**lobby**	0,59	0,76	**embellish**	0,74	0,67
**hilarious**	0,57	0,68	**buffoon**	0,71	0,71	**mine**	0,57	0,74	**encumber**	0,69	0,72
**indicate**	0,55	0,69	**burly**	0,58	0,65	**orchard**	0,52	0,72	**fissure**	0,62	0,69
**launch**	0,57	0,75	**cleave**	0,6	0,67	**peak**	0,58	0,66	**hodgepodge**	0,78	0,73
**marsh**	0,59	0,68	**domicile**	0,64	0,68	**prohibit**	0,54	0,7	**lassitude**	0,63	0,77
**mend**	0,61	0,75	**horizontal**	0,74	0,72	**scorched**	0,82	0,79	**malefactor**	0,88	0,79
**quarrel**	0,55	0,6	**jowl**	0,6	0,63	**shatter**	0,52	0,73	**matron**	0,86	0,81
**royal**	0,52	0,74	**knoll**	0,65	0,69	**transparent**	0,64	0,71	**melancholy**	0,69	0,69
**souvenir**	0,56	0,73	**ledger**	0,59	0,65	**trinket**	0,81	0,73	**onerous**	0,57	0,72
**stubborn**	0,54	0,76	**lethargic**	0,61	0,66	**tutor**	0,52	0,74	**prone**	0,7	0,71
**absorb**	0,58	0,74	**orifice**	0,65	0,67	**utensil**	0,53	0,75	**revel**	0,63	0,64
**antenna**	0,52	0,74	**pennant**	0,68	0,72	**boulevard**	0,54	0,74	**scintillating**	0,67	0,68
**blueprint**	0,6	0,72	**precipice**	0,67	0,66	**debris**	0,59	0,71	**surly**	0,63	0,66
**carriage**	0,53	0,71	**spry**	0,72	0,73	**discouraged**	0,56	0,73	**atoll**	0,57	0,63
**citrus**	0,55	0,7	**wallowing**	0,59	0,67	**drought**	0,54	0,72	**cataract**	0,67	0,81
**diagram**	0,55	0,76	**alms**	0,7	0,7	**fragment**	0,55	0,7	**disparate**	0,65	0,79
**dissolved**	0,53	0,75	**balustrade**	0,59	0,65	**hurl**	0,55	0,6	**ebullience**	0,56	0,72
**elastic**	0,55	0,71	**ellipse**	0,74	0,74	**judiciary**	0,53	0,71	**flaccid**	0,67	0,71
**engraved**	0,52	0,74	**glower**	0,92	0,76	**morsel**	0,72	0,69	**hirsute**	0,56	0,74
**festive**	0,53	0,71	**helix**	0,73	0,7	**pillars**	0,54	0,74	**hovel**	0,56	0,6
**herd**	0,52	0,74	**indisposed**	0,63	0,64	**reap**	0,57	0,62	**inclement**	0,62	0,7
**crossroads**	0,52	0,72	**iridescent**	0,77	0,77	**residential**	0,59	0,78	**infuse**	0,55	0,6
**lotion**	0,52	0,74	**mirth**	0,62	0,67	**slit**	0,62	0,69	**obstreperous**	0,72	0,72
**luggage**	0,51	0,7	**opulent**	0,59	0,64	**snarl**	0,54	0,72	**paucity**	0,61	0,73
**pamphlet**	0,58	0,73	**parry**	0,66	0,68	**stampede**	0,52	0,72	**pecuniary**	0,59	0,74
**quartet**	0,54	0,72	**perusal**	0,66	0,74	**startled**	0,52	0,66	**plethora**	0,85	0,75
**ripple**	0,51	0,73	**quadruped**	0,82	0,81	**submerged**	0,61	0,74	**potable**	0,65	0,79
**sapling**	0,57	0,66	**quagmire**	0,57	0,61	**weld**	0,52	0,65	**prodigious**	0,74	0,87
**sculpture**	0,51	0,71	**recess**	0,69	0,7	**barricade**	0,54	0,69	**progeny**	0,67	0,77
**transport**	0,57	0,76	**ruminate**	0,59	0,65	**bureau**	0,73	0,7	**repast**	0,58	0,72
**trophy**	0,6	0,75	**shorn**	0,64	0,67	**congestion**	0,53	0,61	**sinuous**	0,69	0,71
**vineyard**	0,56	0,63	**trend**	0,66	0,71	**consume**	0,58	0,68	**sumptuous**	0,62	0,68
**cove**	0,56	0,7	**truncate**	0,74	0,85	hangar	0,52	0,74	**detritus**	0,55	0,7
**descend**	0,61	0,62	**abrade**	0,68	0,8	memento	0,7	0,7	**diadem**	0,7	0,82
**desolate**	0,52	0,65	**anomalous**	0,78	0,74	monarch	0,53	0,58	**egress**	0,6	0,75
**dune**	0,56	0,72	**bucolic**	0,76	0,87	shabby	0,53	0,73	**feral**	0,66	0,66
**ensemble**	0,79	0,76	**buffet**	0,79	0,92	tranquil	0,56	0,64	**sustenance**	0,71	0,69
**histrionic**	0,61	0,75	**delineation**	0,7	0,71	acoustic	0,7	0,66	**tractable**	0,69	0,8
**natty**	0,56	0,72	**fecund**	0,67	0,82	brawny	0,6	0,74	**concomitant**	0,74	0,72

Table A4 Feature selection of FA dataset when predicting AS. (P-2) linear regression models were trained (with P equal to the number of features in the dataset) using 5-fold cross validation and at each iteration the feature with the lowest correlation with AS was removed from the regressors. The mean R^2 in the train and test set of each model across the folds, as well as the name of the feature dropped at each iteration, is reported in the Table.

Model Number	Features dropped	AS	
		Test R2 (mean ± std)	Train R2 (mean ± std)
**c**	**FA in tract cingulate gyrus part of cingulum right**	**0.009±0.003**	**0.012±0.001**
1	FA in tract acoustic radiation right	0.009±0.003	0.012±0.001
2	FA in tract forceps major	0.009±0.003	0.011±0.001
3	FA in tract middle cerebellar peduncle	0.009±0.003	0.011±0.001
4	FA in tract cingulate gyrus part of cingulum left	0.009±0.003	0.011±0.001
5	FA in tract acoustic radiation left	0.009±0.003	0.011±0.001
6	FA in tract inferior fronto occipital fasciculus left	0.009±0.003	0.011±0.001
7	FA in tract forceps minor	0.008±0.003	0.011±0.001
8	FA in tract inferior longitudinal fasciculus left	0.008±0.003	0.01±0.001
9	FA in tract inferior fronto occipital fasciculus right	0.008±0.003	0.01±0.001
10	FA in tract parahippocampal part of cingulum left	0.008±0.003	0.009±0.001
11	FA in tract inferior longitudinal fasciculus right	0.008±0.003	0.009±0.001
12	FA in tract superior longitudinal fasciculus left	0.007±0.004	0.009±0.001
13	FA in tract posterior thalamic radiation left	0.007±0.003	0.009±0.001
14	FA in tract superior longitudinal fasciculus right	0.007±0.003	0.009±0.001
15	FA in tract corticospinal tract left	0.007±0.003	0.009±0.001
16	FA in tract parahippocampal part of cingulum right	0.007±0.003	0.008±0.001
17	FA in tract anterior thalamic radiation right	0.007±0.003	0.008±0.001
18	FA in tract posterior thalamic radiation right	0.007±0.003	0.008±0.001
19	FA in tract corticospinal tract right	0.007±0.003	0.008±0.001
20	FA in tract uncinate fasciculus left	0.007±0.003	0.008±0.001
21	FA in tract anterior thalamic radiation left	0.007±0.003	0.007±0.001
22	FA in tract medial lemniscus left	0.006±0.003	0.007±0.001
23	FA in tract superior thalamic radiation right	0.006±0.003	0.006±0.001
24	FA in tract superior thalamic radiation left	0.006±0.002	0.006±0.0

Table A5 Feature selection of thickness dataset when predicting AS. (P-2) linear regression models were trained (with P equal to the number of features in the dataset) using 5-fold cross validation and at each iteration the feature with the lowest correlation with AS was removed from the regressors. The mean R^2 in the train and test set of each model across the folds, as well as the name of the feature dropped at each iteration, is reported in the Table.

Model Number	Features dropped	AS	
		Test R2 (mean ± std)	Train R2 (mean ± std)
**0**	**Mean thickness of parstriangularis right hemisphere**	**0.01±0.008**	**0.017±0.002**
1	Mean thickness of caudalanteriorcingulate left hemisphere	0.01±0.008	0.016±0.002
2	Mean thickness of pericalcarine right hemisphere	0.01±0.008	0.016±0.002
3	Mean thickness of superiorfrontal left hemisphere	0.01±0.008	0.016±0.002
4	Mean thickness of lateraloccipital left hemisphere	0.01±0.008	0.016±0.002
5	Mean thickness of parahippocampal right hemisphere	0.009±0.008	0.016±0.002
6	Mean thickness of medialorbitofrontal right hemisphere	0.009±0.008	0.016±0.002
7	Mean thickness of lateraloccipital right hemisphere	0.009±0.008	0.015±0.002
8	Mean thickness of rostralmiddlefrontal right hemisphere	0.009±0.008	0.015±0.002
9	Mean thickness of isthmuscingulate right hemisphere	0.009±0.008	0.015±0.002
10	Mean thickness of paracentral right hemisphere	0.009±0.008	0.015±0.002
11	Mean thickness of medialorbitofrontal left hemisphere	0.009±0.008	0.015±0.002
12	Mean thickness of superiorfrontal right hemisphere	0.009±0.008	0.015±0.002
13	Mean thickness of lateralorbitofrontal right hemisphere	0.008±0.007	0.013±0.002
14	Mean thickness of pericalcarine left hemisphere	0.007±0.007	0.013±0.002
15	Mean thickness of rostralanteriorcingulate left hemisphere	0.007±0.007	0.012±0.002
16	Mean thickness of isthmuscingulate left hemisphere	0.007±0.007	0.012±0.002
17	Mean thickness of inferiortemporal right hemisphere	0.006±0.007	0.012±0.002
18	Mean thickness of posteriorcingulate left hemisphere	0.006±0.007	0.012±0.002
19	Mean thickness of lateralorbitofrontal left hemisphere	0.006±0.007	0.011±0.002
20	Mean thickness of fusiform right hemisphere	0.006±0.007	0.011±0.002
21	Mean thickness of inferiortemporal left hemisphere	0.006±0.007	0.011±0.002
22	Mean thickness of inferiorparietal left hemisphere	0.006±0.007	0.011±0.002
23	Mean thickness of superiorparietal right hemisphere	0.006±0.007	0.01±0.002
24	Mean thickness of entorhinal right hemisphere	0.006±0.006	0.01±0.001
25	Mean thickness of lingual right hemisphere	0.006±0.007	0.01±0.002
26	Mean thickness of posteriorcingulate right hemisphere	0.006±0.006	0.01±0.001
27	Mean thickness of fusiform left hemisphere	0.006±0.005	0.01±0.001
28	Mean thickness of parstriangularis left hemisphere	0.006±0.005	0.009±0.001
29	Mean thickness of inferiorparietal right hemisphere	0.006±0.005	0.009±0.001
30	Mean thickness of caudalanteriorcingulate right hemisphere	0.006±0.005	0.009±0.001
31	Mean thickness of paracentral left hemisphere	0.005±0.005	0.009±0.001
32	Mean thickness of precuneus right hemisphere	0.005±0.005	0.008±0.001
33	Mean thickness of cuneus right hemisphere	0.005±0.005	0.008±0.001
34	Mean thickness of parahippocampal left hemisphere	0.005±0.005	0.008±0.001
35	Mean thickness of superiorparietal left hemisphere	0.005±0.005	0.008±0.001
36	Mean thickness of cuneus left hemisphere	0.005±0.005	0.008±0.001
37	Mean thickness of rostralmiddlefrontal left hemisphere	0.005±0.005	0.008±0.001
38	Mean thickness of rostralanteriorcingulate right hemisphere	0.005±0.005	0.008±0.001
39	Mean thickness of parsorbitalis left hemisphere	0.004±0.004	0.006±0.001
40	Mean thickness of postcentral right hemisphere	0.004±0.004	0.006±0.001
41	Mean thickness of parsorbitalis right hemisphere	0.004±0.004	0.006±0.001
42	Mean thickness of middletemporal left hemisphere	0.004±0.004	0.006±0.001
43	Mean thickness of middletemporal right hemisphere	0.004±0.003	0.006±0.001
44	Mean thickness of supramarginal left hemisphere	0.004±0.003	0.006±0.001
45	Mean thickness of precuneus left hemisphere	0.004±0.003	0.005±0.001
46	Mean thickness of caudalmiddlefrontal right hemisphere	0.003±0.003	0.005±0.001
47	Mean thickness of supramarginal right hemisphere	0.004±0.003	0.005±0.001
48	Mean thickness of lingual left hemisphere	0.003±0.003	0.005±0.001
49	Mean thickness of postcentral left hemisphere	0.004±0.003	0.005±0.001
50	Mean thickness of precentral right hemisphere	0.003±0.004	0.005±0.001
51	Mean thickness of entorhinal left hemisphere	0.003±0.004	0.005±0.001
52	Mean thickness of transversetemporal right hemisphere	0.003±0.003	0.005±0.001
53	Mean thickness of parsopercularis right hemisphere	0.003±0.003	0.005±0.001
54	Mean thickness of caudalmiddlefrontal left hemisphere	0.003±0.003	0.005±0.001
55	Mean thickness of precentral left hemisphere	0.003±0.003	0.005±0.001
56	Mean thickness of parsopercularis left hemisphere	0.004±0.003	0.004±0.001
57	Mean thickness of transversetemporal left hemisphere	0.004±0.003	0.004±0.001
58	Mean thickness of superiortemporal right hemisphere	0.003±0.003	0.004±0.001

Table A6 Feature selection of intensity dataset when predicting AS. (P-2) linear regression models were trained (with P equal to the number of features in the dataset) using 5-fold cross validation and at each iteration the feature with the lowest correlation with AS was removed from the regressors. The mean R^2 in the train and test set of each model across the folds, as well as the name of the feature dropped at each iteration, is reported in the Table

**Model Number**	**Features dropped**	**AS**	
		**Test R2 (mean ± std)**	**Train R2 (mean ± std)**
**0**	Mean intensity of CSF whole brain	0.008±0.007	0.014±0.002
1	Mean intensity of non WM hypointensities whole brain	0.008±0.007	0.014±0.002
2	Mean intensity of CC Mid Posterior whole brain	0.008±0.007	0.014±0.002
3	Mean intensity of 5th Ventricle whole brain	0.008±0.006	0.014±0.002
4	Mean intensity of Cerebellum White Matter left hemisphere	0.008±0.006	0.014±0.002
5	Mean intensity of CC Posterior whole brain	0.008±0.006	0.014±0.002
6	Volume of WM hypointensities whole brain	0.008±0.006	0.014±0.002
7	Mean intensity of Cerebellum Cortex right hemisphere	0.009±0.006	0.014±0.002
8	Mean intensity of Amygdala left hemisphere	0.009±0.006	0.014±0.002
9	Mean intensity of WM hypointensities whole brain	0.009±0.006	0.014±0.002
10	Mean intensity of Cerebellum Cortex left hemisphere	0.009±0.006	0.014±0.002
**11**	**Mean intensity of Cerebellum White Matter right hemisphere**	**0.009±0.006**	**0.014±0.002**
12	Volume of non WM hypointensities whole brain	0.009±0.006	0.013±0.002
13	Mean intensity of Accumbens area right hemisphere	0.008±0.006	0.013±0.001
14	Mean intensity of vessel left hemisphere	0.008±0.006	0.013±0.001
15	Mean intensity of Amygdala right hemisphere	0.008±0.006	0.013±0.001
16	Mean intensity of vessel right hemisphere	0.007±0.006	0.011±0.001
17	Mean intensity of Inf Lat Vent right hemisphere	0.007±0.006	0.011±0.002
18	Mean intensity of 4th Ventricle whole brain	0.007±0.006	0.011±0.001
19	Mean intensity of Inf Lat Vent left hemisphere	0.006±0.006	0.01±0.001
20	Mean intensity of CC Central whole brain	0.006±0.005	0.01±0.001
21	Mean intensity of Caudate right hemisphere	0.006±0.006	0.01±0.001
22	Mean intensity of Optic Chiasm whole brain	0.006±0.006	0.01±0.001
23	Mean intensity of Hippocampus left hemisphere	0.006±0.006	0.01±0.001
24	Mean intensity of Lateral Ventricle left hemisphere	0.006±0.006	0.01±0.001
25	Mean intensity of 3rd Ventricle whole brain	0.006±0.006	0.01±0.001
26	Mean intensity of Hippocampus right hemisphere	0.006±0.005	0.009±0.001
27	Mean intensity of Lateral Ventricle right hemisphere	0.006±0.005	0.009±0.001
28	Mean intensity of VentralDC right hemisphere	0.007±0.005	0.009±0.001
29	Mean intensity of VentralDC left hemisphere	0.007±0.005	0.009±0.001
30	Mean intensity of Caudate left hemisphere	0.006±0.005	0.008±0.001
31	Mean intensity of Accumbens area left hemisphere	0.006±0.005	0.008±0.001
32	Mean intensity of choroid plexus left hemisphere	0.006±0.005	0.008±0.001
33	Mean intensity of Putamen right hemisphere	0.006±0.005	0.008±0.001
34	Mean intensity of Brain Stem whole brain	0.006±0.005	0.008±0.001
35	Mean intensity of Pallidum right hemisphere	0.006±0.005	0.008±0.001
36	Mean intensity of CC Anterior whole brain	0.007±0.005	0.008±0.001
37	Mean intensity of CC Mid Anterior whole brain	0.006±0.004	0.007±0.001
38	Mean intensity of Pallidum left hemisphere	0.005±0.004	0.006±0.001
39	Mean intensity of choroid plexus right hemisphere	0.005±0.004	0.006±0.001
40	Mean intensity of Putamen left hemisphere	0.002±0.003	0.003±0.001
41	Mean intensity of Thalamus Proper left hemisphere	0.002±0.003	0.003±0.001

Table A7 Feature selection of volume dataset when predicting AS. (P-2) linear regression models were trained (with P equal to the number of features in the dataset) using 5-fold cross validation and at each iteration the feature with the lowest correlation with AS was removed from the regressors. The mean R^2 in the train and test set of each model across the folds, as well as the name of the feature dropped at each iteration, is reported in the Table.

Model Number	Features dropped	AS	
		**Test R2 (mean ± std)**	**Train R2 (mean ± std)**
0	Volume of grey matter in Crus I Cerebellum vermis	0.025±0.009	0.041±0.002
**1**	**Volume of grey matter in Brain Stem**	**0.025±0.008**	**0.041±0.002**
2	Volume of grey matter in X Cerebellum vermis	0.023±0.008	0.039±0.002
3	Volume of grey matter in Pallidum left	0.023±0.008	0.039±0.002
4	Volume of grey matter in Pallidum right	0.023±0.008	0.039±0.002
5	Volume of grey matter in Caudate left	0.023±0.008	0.039±0.002
6	Volume of grey matter in Inferior Temporal Gyrus posterior division left	0.023±0.008	0.039±0.002
7	Volume of grey matter in Inferior Frontal Gyrus pars triangularis left	0.023±0.008	0.038±0.002
8	Volume of grey matter in Occipital Pole right	0.023±0.008	0.038±0.002
9	Volume of grey matter in Juxtapositional Lobule Cortex right	0.022±0.008	0.038±0.002
10	Volume of grey matter in Cingulate Gyrus anterior division left	0.022±0.007	0.038±0.002
11	Volume of grey matter in I IV Cerebellum right	0.022±0.008	0.037±0.002
12	Volume of grey matter in Putamen left	0.022±0.008	0.037±0.002
13	Volume of grey matter in Cuneal Cortex left	0.022±0.008	0.037±0.002
14	Volume of grey matter in Caudate right	0.022±0.008	0.037±0.002
15	Volume of grey matter in Occipital Pole left	0.022±0.008	0.037±0.002
16	Volume of grey matter in Inferior Temporal Gyrus anterior division left	0.021±0.007	0.036±0.002
17	Volume of grey matter in Inferior Temporal Gyrus anterior division right	0.021±0.007	0.036±0.002
18	Volume of grey matter in Inferior Temporal Gyrus temporooccipital part left	0.021±0.007	0.036±0.002
19	Volume of grey matter in Cingulate Gyrus anterior division right	0.021±0.007	0.036±0.002
20	Volume of grey matter in I IV Cerebellum left	0.021±0.007	0.036±0.002
21	Volume of grey matter in Supracalcarine Cortex left	0.021±0.007	0.035±0.002
22	Volume of grey matter in Intracalcarine Cortex left	0.021±0.007	0.035±0.002
23	Volume of grey matter in Putamen right	0.021±0.007	0.035±0.002
24	Volume of grey matter in Inferior Frontal Gyrus pars opercularis right	0.021±0.007	0.035±0.002
25	Volume of grey matter in Inferior Frontal Gyrus pars triangularis right	0.021±0.007	0.035±0.002
26	Volume of grey matter in Superior Parietal Lobule left	0.021±0.007	0.035±0.002
27	Volume of grey matter in Juxtapositional Lobule Cortex left	0.021±0.007	0.035±0.002
28	Volume of grey matter in Heschl s Gyrus includes H1 and H2 left	0.021±0.007	0.034±0.002
29	Volume of grey matter in Supramarginal Gyrus anterior division right	0.021±0.007	0.034±0.002
30	Volume of grey matter in X Cerebellum right	0.021±0.007	0.034±0.002
31	Volume of grey matter in X Cerebellum left	0.021±0.007	0.034±0.002
32	Volume of grey matter in Inferior Temporal Gyrus temporooccipital part right	0.021±0.007	0.034±0.002
33	Volume of grey matter in Superior Parietal Lobule right	0.021±0.007	0.034±0.002
34	Volume of grey matter in Supramarginal Gyrus anterior division left	0.021±0.007	0.034±0.002
35	Volume of grey matter in VI Cerebellum vermis	0.021±0.007	0.034±0.002
36	Volume of grey matter in Middle Temporal Gyrus temporooccipital part left	0.021±0.007	0.034±0.002
37	Volume of grey matter in VIIb Cerebellum vermis	0.021±0.007	0.034±0.002
38	Volume of grey matter in Supramarginal Gyrus posterior division left	0.021±0.006	0.034±0.002
39	Volume of grey matter in Inferior Temporal Gyrus posterior division right	0.022±0.006	0.034±0.002
40	Volume of grey matter in Occipital Fusiform Gyrus left	0.021±0.007	0.033±0.002
41	Volume of grey matter in Frontal Operculum Cortex left	0.021±0.007	0.033±0.002
42	Volume of grey matter in Middle Temporal Gyrus temporooccipital part right	0.021±0.006	0.033±0.002
43	Volume of grey matter in Crus II Cerebellum vermis	0.021±0.006	0.033±0.002
44	Volume of grey matter in Inferior Frontal Gyrus pars opercularis left	0.021±0.006	0.033±0.002
45	Volume of grey matter in Intracalcarine Cortex right	0.021±0.006	0.033±0.002
46	Volume of grey matter in Paracingulate Gyrus right	0.021±0.006	0.033±0.002
47	Volume of grey matter in Angular Gyrus right	0.021±0.006	0.032±0.001
48	Volume of grey matter in Parietal Operculum Cortex left	0.021±0.006	0.032±0.001
49	Volume of grey matter in Thalamus left	0.021±0.006	0.032±0.002
50	Volume of grey matter in IX Cerebellum right	0.021±0.006	0.032±0.002
51	Volume of grey matter in Cuneal Cortex right	0.021±0.006	0.032±0.002
52	Volume of grey matter in Planum Temporale left	0.021±0.006	0.032±0.002
53	Volume of grey matter in Parietal Operculum Cortex right	0.021±0.007	0.032±0.002
54	Volume of grey matter in Parahippocampal Gyrus posterior division left	0.021±0.007	0.031±0.002
55	Volume of grey matter in VIIIb Cerebellum vermis	0.021±0.007	0.031±0.002
56	Volume of grey matter in Temporal Occipital Fusiform Cortex left	0.021±0.006	0.031±0.002
57	Volume of grey matter in Thalamus right	0.021±0.006	0.031±0.002
58	Volume of grey matter in Supramarginal Gyrus posterior division right	0.021±0.006	0.031±0.002
59	Volume of grey matter in Angular Gyrus left	0.021±0.006	0.031±0.001
60	Volume of grey matter in Frontal Medial Cortex left	0.021±0.006	0.03±0.001
61	Volume of grey matter in IX Cerebellum left	0.02±0.006	0.03±0.001
62	Volume of grey matter in VIIIb Cerebellum right	0.02±0.006	0.03±0.001
63	Volume of grey matter in Frontal Operculum Cortex right	0.02±0.005	0.03±0.001
64	Volume of grey matter in Ventral Striatum left	0.02±0.005	0.03±0.001
65	Volume of grey matter in Middle Temporal Gyrus anterior division right	0.02±0.005	0.029±0.001
66	Volume of grey matter in Parahippocampal Gyrus posterior division right	0.02±0.005	0.029±0.001

67	Volume of grey matter in Middle Frontal Gyrus right	0.02±0.005	0.029±0.001
68	Volume of grey matter in Supracalcarine Cortex right	0.02±0.005	0.029±0.001
69	Volume of grey matter in Crus I Cerebellum right	0.02±0.005	0.029±0.001
70	Volume of grey matter in Superior Frontal Gyrus left	0.02±0.005	0.029±0.001
71	Volume of grey matter in Paracingulate Gyrus left	0.02±0.005	0.029±0.001
72	Volume of grey matter in Crus I Cerebellum left	0.02±0.005	0.029±0.001
73	Volume of grey matter in Middle Frontal Gyrus left	0.02±0.006	0.028±0.001
74	Volume of grey matter in Occipital Fusiform Gyrus right	0.02±0.006	0.028±0.001
75	Volume of grey matter in IX Cerebellum vermis	0.02±0.006	0.028±0.001
76	Volume of grey matter in Lateral Occipital Cortex inferior division left	0.02±0.006	0.028±0.001
77	Volume of grey matter in Middle Temporal Gyrus posterior division right	0.02±0.006	0.028±0.001
78	Volume of grey matter in Superior Frontal Gyrus right	0.02±0.006	0.028±0.001
79	Volume of grey matter in Temporal Occipital Fusiform Cortex right	0.021±0.005	0.028±0.001
80	Volume of grey matter in Precuneous Cortex right	0.021±0.005	0.028±0.001
81	Volume of grey matter in Central Opercular Cortex right	0.021±0.006	0.028±0.001
82	Volume of grey matter in Lateral Occipital Cortex superior division right	0.02±0.005	0.027±0.001
83	Volume of grey matter in Precuneous Cortex left	0.02±0.005	0.027±0.001
84	Volume of grey matter in VIIIa Cerebellum vermis	0.02±0.006	0.027±0.001
85	Volume of grey matter in Lateral Occipital Cortex inferior division right	0.02±0.006	0.027±0.001
86	Volume of grey matter in Middle Temporal Gyrus anterior division left	0.02±0.005	0.027±0.001
87	Volume of grey matter in Heschl s Gyrus includes H1 and H2 right	0.02±0.006	0.027±0.001
88	Volume of grey matter in Frontal Medial Cortex right	0.02±0.006	0.027±0.001
89	Volume of grey matter in V Cerebellum right	0.02±0.006	0.026±0.001
90	Volume of grey matter in Frontal Orbital Cortex right	0.02±0.006	0.026±0.001
91	Volume of grey matter in Temporal Fusiform Cortex posterior division left	0.02±0.006	0.026±0.002
92	Volume of grey matter in Middle Temporal Gyrus posterior division left	0.02±0.006	0.026±0.001
93	Volume of grey matter in Postcentral Gyrus right	0.02±0.006	0.026±0.001
94	Volume of grey matter in VIIIb Cerebellum left	0.02±0.006	0.026±0.001
95	Volume of grey matter in V Cerebellum left	0.02±0.006	0.026±0.001
96	Volume of grey matter in Postcentral Gyrus left	0.02±0.006	0.026±0.001
97	Volume of grey matter in Temporal Fusiform Cortex anterior division right	0.02±0.006	0.025±0.001
98	Volume of grey matter in Superior Temporal Gyrus anterior division right	0.02±0.006	0.025±0.001
99	Volume of grey matter in Ventral Striatum right	0.02±0.006	0.025±0.001
100	Volume of grey matter in Precentral Gyrus right	0.02±0.006	0.025±0.001
101	Volume of grey matter in Lingual Gyrus left	0.02±0.006	0.025±0.001
102	Volume of grey matter in Subcallosal Cortex right	0.02±0.006	0.025±0.002
103	Volume of grey matter in Planum Polare right	0.02±0.006	0.025±0.002
104	Volume of grey matter in Cingulate Gyrus posterior division left	0.02±0.006	0.025±0.002
105	Volume of grey matter in Cingulate Gyrus posterior division right	0.02±0.006	0.025±0.002
106	Volume of grey matter in Superior Temporal Gyrus posterior division left	0.02±0.006	0.025±0.002
107	Volume of grey matter in Subcallosal Cortex left	0.02±0.006	0.025±0.002
108	Volume of grey matter in Parahippocampal Gyrus anterior division right	0.02±0.006	0.024±0.002
109	Volume of grey matter in Temporal Fusiform Cortex posterior division right	0.02±0.006	0.024±0.002
110	Volume of grey matter in Frontal Orbital Cortex left	0.02±0.006	0.024±0.002
111	Volume of grey matter in Lateral Occipital Cortex superior division left	0.02±0.006	0.024±0.002
112	Volume of grey matter in Lingual Gyrus right	0.02±0.006	0.024±0.002
113	Volume of grey matter in Precentral Gyrus left	0.02±0.006	0.024±0.002
114	Volume of grey matter in VIIIa Cerebellum left	0.02±0.007	0.024±0.002
115	Volume of grey matter in Superior Temporal Gyrus posterior division right	0.021±0.007	0.024±0.002
116	Volume of grey matter in Parahippocampal Gyrus anterior division left	0.021±0.007	0.024±0.002
117	Volume of grey matter in Planum Temporale right	0.021±0.007	0.024±0.002
118	Volume of grey matter in VIIIa Cerebellum right	0.021±0.007	0.024±0.002
119	Volume of grey matter in Temporal Pole left	0.021±0.007	0.024±0.002
120	Volume of grey matter in Temporal Pole right	0.021±0.007	0.024±0.002
121	Volume of grey matter in Planum Polare left	0.021±0.007	0.024±0.002
122	Volume of grey matter in Central Opercular Cortex left	0.021±0.007	0.024±0.002
123	Volume of grey matter in VIIb Cerebellum left	0.021±0.007	0.023±0.002
124	Volume of grey matter in Temporal Fusiform Cortex anterior division left	0.021±0.007	0.023±0.002
125	Volume of grey matter in Insular Cortex left	0.021±0.008	0.023±0.002
126	Volume of grey matter in Superior Temporal Gyrus anterior division left	0.021±0.008	0.022±0.002
127	Volume of grey matter in VI Cerebellum left	0.019±0.007	0.021±0.002
128	Volume of grey matter in Crus II Cerebellum left	0.019±0.007	0.021±0.002
129	Volume of grey matter in VI Cerebellum right	0.02±0.007	0.021±0.002
130	Volume of grey matter in Crus II Cerebellum right	0.019±0.007	0.021±0.002
131	Volume of grey matter in Frontal Pole left	0.019±0.007	0.02±0.002
132	Volume of grey matter in VIIb Cerebellum right	0.019±0.007	0.02±0.002
133	Volume of grey matter in Insular Cortex right	0.017±0.005	0.018±0.001
134	Volume of grey matter in Hippocampus right	0.017±0.005	0.018±0.001
135	Volume of grey matter in Frontal Pole right	0.017±0.005	0.018±0.001
136	Volume of grey matter in Hippocampus left	0.016±0.005	0.016±0.001
137	Volume of grey matter in Amygdala right	0.025±0.009	0.041±0.002

Table A8 Feature selection of FA dataset when predicting DT. (P-2) linear regression models were trained (with P equal to the number of features in the dataset) using 5-fold cross validation and at each iteration the feature with the lowest correlation with DT was removed from the regressors. The mean R^2 in the train and test set of each model across the folds, as well as the name of the feature dropped at each iteration, is reported in the Table.

Model Number	Features dropped	DT	
		Test R2 (mean ± std)	Train R2 (mean ± std)
0	FA in tract superior thalamic radiation left	0.0±0.002	0.004±0.0
1	FA in tract superior thalamic radiation right	0.0±0.002	0.004±0.0
2	FA in tract corticospinal tract left	0.001±0.002	0.004±0.001
3	FA in tract posterior thalamic radiation right	0.001±0.002	0.004±0.0
4	FA in tract corticospinal tract right	0.001±0.002	0.004±0.0
5	FA in tract inferior longitudinal fasciculus left	0.001±0.002	0.004±0.0
6	FA in tract superior longitudinal fasciculus left	0.001±0.002	0.004±0.0
7	FA in tract parahippocampal part of cingulum left	0.001±0.002	0.004±0.0
8	FA in tract middle cerebellar peduncle	0.001±0.002	0.003±0.0
9	FA in tract inferior fronto occipital fasciculus right	0.001±0.002	0.003±0.0
10	FA in tract inferior fronto occipital fasciculus left	0.001±0.002	0.003±0.0
11	FA in tract anterior thalamic radiation left	0.001±0.002	0.003±0.0
**12**	**FA in tract forceps minor**	**0.001±0.002**	**0.003±0.0**
13	FA in tract posterior thalamic radiation left	0.001±0.002	0.003±0.0
14	FA in tract parahippocampal part of cingulum right	0.001±0.001	0.002±0.0
15	FA in tract inferior longitudinal fasciculus right	0.001±0.001	0.002±0.0
16	FA in tract medial lemniscus left	0.001±0.001	0.002±0.0
17	FA in tract anterior thalamic radiation right	0.001±0.001	0.002±0.0
18	FA in tract forceps major	0.001±0.001	0.002±0.0
19	FA in tract superior longitudinal fasciculus right	0.001±0.001	0.002±0.0
20	FA in tract acoustic radiation left	0.001±0.001	0.002±0.0
21	FA in tract medial lemniscus right	0.001±0.001	0.002±0.0
22	FA in tract cingulate gyrus part of cingulum left	0.001±0.0	0.001±0.0
23	FA in tract cingulate gyrus part of cingulum right	0.001±0.0	0.001±0.0
24	FA in tract acoustic radiation right	0.001±0.0	0.001±0.0

Table A9 Feature selection of thickness dataset when predicting DT. (P-2) linear regression models were trained (with P equal to the number of features in the dataset) using 5-fold cross validation and at each iteration the feature with the lowest correlation with DT was removed from the regressors. The mean R^2 in the train and test set of each model across the folds, as well as the name of the feature dropped at each iteration, is reported in the Table.

Model Number	Features dropped	DT	
		Test R2 (mean ± std)	Train R2 (mean ± std)
**0**	Mean thickness of postcentral left hemisphere	-0.001±0.002	0.007±0.001
1	Mean thickness of isthmuscingulate left hemisphere	-0.001±0.003	0.007±0.001
2	Mean thickness of entorhinal right hemisphere	-0.0±0.003	0.007±0.001
3	Mean thickness of postcentral right hemisphere	-0.001±0.003	0.007±0.001
4	Mean thickness of superiorparietal left hemisphere	-0.0±0.003	0.007±0.001
5	Mean thickness of isthmuscingulate right hemisphere	-0.0±0.003	0.007±0.001
6	Mean thickness of paracentral right hemisphere	-0.0±0.003	0.007±0.001
7	Mean thickness of parstriangularis right hemisphere	-0.0±0.003	0.007±0.001
8	Mean thickness of superiorparietal right hemisphere	-0.0±0.003	0.007±0.001
9	Mean thickness of cuneus left hemisphere	-0.001±0.003	0.006±0.001
10	Mean thickness of rostralmiddlefrontal right hemisphere	-0.001±0.003	0.006±0.001
11	Mean thickness of posteriorcingulate right hemisphere	-0.001±0.003	0.006±0.001
12	Mean thickness of parsopercularis left hemisphere	-0.001±0.003	0.006±0.001
13	Mean thickness of parstriangularis left hemisphere	-0.0±0.003	0.006±0.001
14	Mean thickness of rostralanteriorcingulate right hemisphere	-0.0±0.003	0.006±0.001
15	Mean thickness of parsopercularis right hemisphere	-0.0±0.003	0.006±0.001
16	Mean thickness of posteriorcingulate left hemisphere	-0.0±0.003	0.006±0.001
17	Mean thickness of lateraloccipital right hemisphere	-0.0±0.003	0.006±0.001
18	Mean thickness of precuneus right hemisphere	-0.0±0.003	0.005±0.001
19	Mean thickness of lateraloccipital left hemisphere	-0.0±0.003	0.005±0.001
20	Mean thickness of precentral right hemisphere	-0.001±0.003	0.005±0.001
21	Mean thickness of precentral left hemisphere	-0.0±0.003	0.005±0.001
22	Mean thickness of rostralmiddlefrontal left hemisphere	-0.0±0.003	0.005±0.001
23	Mean thickness of caudalmiddlefrontal right hemisphere	-0.0±0.003	0.005±0.001
24	Mean thickness of entorhinal left hemisphere	-0.0±0.003	0.005±0.001
25	Mean thickness of medialorbitofrontal right hemisphere	0.0±0.002	0.005±0.001
26	Mean thickness of caudalmiddlefrontal left hemisphere	0.0±0.003	0.005±0.001
27	Mean thickness of pericalcarine right hemisphere	0.0±0.003	0.005±0.001
28	Mean thickness of transversetemporal left hemisphere	0.001±0.003	0.005±0.001
29	Mean thickness of parsorbitalis right hemisphere	0.001±0.003	0.005±0.001
30	Mean thickness of supramarginal right hemisphere	0.001±0.003	0.005±0.001
31	Mean thickness of lingual right hemisphere	0.001±0.003	0.005±0.001
32	Mean thickness of fusiform left hemisphere	0.001±0.003	0.005±0.001
33	Mean thickness of inferiorparietal right hemisphere	0.001±0.003	0.005±0.001
34	Mean thickness of paracentral left hemisphere	0.001±0.003	0.004±0.001
35	Mean thickness of precuneus left hemisphere	0.001±0.003	0.004±0.001
36	Mean thickness of transversetemporal right hemisphere	0.001±0.003	0.004±0.001
37	Mean thickness of medialorbitofrontal left hemisphere	0.001±0.003	0.004±0.001
38	Mean thickness of lingual left hemisphere	0.001±0.003	0.004±0.001
39	Mean thickness of inferiorparietal left hemisphere	0.001±0.002	0.004±0.001
40	Mean thickness of parahippocampal left hemisphere	0.001±0.002	0.004±0.001
41	Mean thickness of pericalcarine left hemisphere	0.001±0.002	0.004±0.001
42	Mean thickness of superiorfrontal right hemisphere	0.001±0.002	0.004±0.001
43	Mean thickness of supramarginal left hemisphere	0.001±0.002	0.004±0.001
44	Mean thickness of lateralorbitofrontal left hemisphere	0.001±0.002	0.004±0.001
**45**	**Mean thickness of cuneus right hemisphere**	**0.001±0.003**	**0.004±0.001**
46	Mean thickness of superiorfrontal left hemisphere	0.0±0.003	0.002±0.001
47	Mean thickness of parahippocampal right hemisphere	0.0±0.002	0.002±0.001
48	Mean thickness of parsorbitalis left hemisphere	0.0±0.002	0.002±0.001
49	Mean thickness of caudalanteriorcingulate left hemisphere	0.0±0.002	0.002±0.001
50	Mean thickness of fusiform right hemisphere	0.001±0.002	0.002±0.001
51	Mean thickness of rostralanteriorcingulate left hemisphere	0.001±0.002	0.002±0.001
52	Mean thickness of superiortemporal right hemisphere	0.001±0.002	0.002±0.0
53	Mean thickness of caudalanteriorcingulate right hemisphere	0.001±0.002	0.002±0.0
54	Mean thickness of superiortemporal left hemisphere	0.001±0.002	0.002±0.0
55	Mean thickness of middletemporal right hemisphere	0.001±0.002	0.002±0.001
56	Mean thickness of lateralorbitofrontal right hemisphere	0.001±0.002	0.002±0.001
57	Mean thickness of inferiortemporal right hemisphere	0.001±0.002	0.002±0.0
58	Mean thickness of inferiortemporal left hemisphere	0.001±0.002	0.001±0.0

Table A10 Feature selection of intensity dataset when predicting DT. (P-2) linear regression models were trained (with P equal to the number of features in the dataset) using 5-fold cross validation and at each iteration the feature with the lowest correlation with DT was removed from the regressors. The mean R^2 in the train and test set of each model across the folds, as well as the name of the feature dropped at each iteration, is reported in the Table.

**Model Number**	**Features dropped**	**DT**	
		**Test R2 (mean ± std)**	**Train R2 (mean ± std)**
**0**	Mean intensity of Accumbens area right hemisphere	-0.0±0.002	0.005±0.001
1	Mean intensity of Cerebellum White Matter right hemisphere	-0.001±0.002	0.005±0.001
2	Mean intensity of Caudate left hemisphere	-0.001±0.002	0.005±0.001
3	Mean intensity of Caudate right hemisphere	-0.001±0.002	0.005±0.001
4	Mean intensity of Inf Lat Vent left hemisphere	-0.001±0.003	0.004±0.001
5	Mean intensity of Inf Lat Vent right hemisphere	-0.001±0.002	0.004±0.001
6	Mean intensity of WM hypointensities whole brain	-0.001±0.002	0.004±0.0
7	Mean intensity of Putamen left hemisphere	-0.001±0.001	0.004±0.0
8	Mean intensity of 5th Ventricle whole brain	-0.001±0.001	0.004±0.0
9	Mean intensity of vessel right hemisphere	-0.001±0.001	0.004±0.0
10	Mean intensity of choroid plexus right hemisphere	-0.0±0.001	0.004±0.0
**11**	Mean intensity of CC Mid Anterior whole brain	-0.0±0.001	0.004±0.0
12	Mean intensity of Pallidum right hemisphere	-0.0±0.001	0.004±0.0
13	Mean intensity of non WM hypointensities whole brain	-0.0±0.001	0.004±0.0
14	Mean intensity of choroid plexus left hemisphere	-0.0±0.001	0.004±0.0
15	Volume of non WM hypointensities whole brain	-0.0±0.001	0.004±0.0
16	Mean intensity of Putamen right hemisphere	-0.0±0.001	0.003±0.0
17	Mean intensity of Cerebellum White Matter left hemisphere	-0.001±0.001	0.003±0.0
18	Mean intensity of Pallidum left hemisphere	-0.001±0.001	0.003±0.0
19	Mean intensity of CC Posterior whole brain	-0.001±0.001	0.003±0.0
20	Mean intensity of Optic Chiasm whole brain	-0.0±0.001	0.003±0.0
21	Mean intensity of CC Anterior whole brain	-0.0±0.001	0.003±0.0
22	Mean intensity of Thalamus Proper left hemisphere	-0.0±0.001	0.003±0.0
23	Mean intensity of vessel left hemisphere	-0.001±0.001	0.003±0.0
24	Volume of WM hypointensities whole brain	-0.0±0.001	0.002±0.0
25	Mean intensity of VentralDC left hemisphere	-0.0±0.001	0.002±0.0
26	Mean intensity of Amygdala right hemisphere	0.0±0.001	0.002±0.0
27	Mean intensity of Brain Stem whole brain	0.0±0.001	0.002±0.0
28	Mean intensity of VentralDC right hemisphere	0.0±0.001	0.002±0.0
29	Mean intensity of Thalamus Proper right hemisphere	0.0±0.001	0.002±0.0
30	Mean intensity of CC Central whole brain	0.0±0.001	0.002±0.0
31	Mean intensity of CC Mid Posterior whole brain	0.0±0.001	0.002±0.0
32	Mean intensity of Cerebellum Cortex right hemisphere	0.0±0.001	0.002±0.0
33	Mean intensity of Accumbens area left hemisphere	-0.0±0.001	0.001±0.0
34	Mean intensity of Lateral Ventricle left hemisphere	0.0±0.001	0.001±0.0
35	Mean intensity of Lateral Ventricle right hemisphere	0.0±0.001	0.001±0.0
36	Mean intensity of Amygdala left hemisphere	0.0±0.001	0.001±0.0
37	Mean intensity of CSF whole brain	0.001±0.001	0.001±0.0
38	Mean intensity of Cerebellum Cortex left hemisphere	0.001±0.001	0.001±0.0
39	Mean intensity of 3rd Ventricle whole brain	0.001±0.001	0.001±0.0
40	Mean intensity of Hippocampus left hemisphere	0.001±0.001	0.001±0.0
**41**	**Mean intensity of 4th Ventricle whole brain**	**0.001±0.001**	**0.001±0.0**

Table A11 Feature selection of volume dataset when predicting AS. (P-2) linear regression models were trained (with P equal to the number of features in the dataset) using 5-fold cross validation and at each iteration the feature with the lowest correlation with DT was removed from the regressors. The mean R^2 in the train and test set of each model across the folds, as well as the name of the feature dropped at each iteration, is reported in the Table.

Model Number	Features dropped	DT	
		**Test R2 (mean ± std)**	**Train R2 (mean ± std)**
0	Volume of grey matter in Hippocampus left	-0.003±0.004	0.015±0.001
1	Volume of grey matter in Planum Polare right	-0.002±0.004	0.015±0.001
2	Volume of grey matter in Inferior Temporal Gyrus temporooccipital part right	-0.002±0.005	0.015±0.001
3	Volume of grey matter in Lateral Occipital Cortex inferior division left	-0.002±0.004	0.015±0.001
4	Volume of grey matter in Inferior Frontal Gyrus pars triangularis right	-0.002±0.004	0.015±0.001
5	Volume of grey matter in Caudate right	-0.002±0.004	0.015±0.001
6	Volume of grey matter in Middle Temporal Gyrus anterior division left	-0.002±0.004	0.015±0.001
7	Volume of grey matter in Temporal Fusiform Cortex posterior division left	-0.002±0.004	0.015±0.001
8	Volume of grey matter in Planum Temporale left	-0.002±0.004	0.014±0.001
9	Volume of grey matter in Cingulate Gyrus anterior division left	-0.002±0.004	0.014±0.001
10	Volume of grey matter in Supramarginal Gyrus anterior division left	-0.002±0.004	0.014±0.001
11	Volume of grey matter in Caudate left	-0.002±0.004	0.014±0.001
12	Volume of grey matter in Cingulate Gyrus posterior division left	-0.002±0.004	0.014±0.001
13	Volume of grey matter in Supramarginal Gyrus posterior division right	-0.002±0.004	0.014±0.001
14	Volume of grey matter in Supramarginal Gyrus anterior division right	-0.002±0.004	0.014±0.001
15	Volume of grey matter in Middle Temporal Gyrus anterior division right	-0.002±0.004	0.014±0.001
16	Volume of grey matter in Subcallosal Cortex left	-0.002±0.004	0.014±0.001
17	Volume of grey matter in Superior Temporal Gyrus anterior division left	-0.002±0.004	0.014±0.001
18	Volume of grey matter in Planum Polare left	-0.002±0.004	0.014±0.001
19	Volume of grey matter in Juxtapositional Lobule Cortex right	-0.002±0.004	0.014±0.001
20	Volume of grey matter in Lateral Occipital Cortex inferior division right	-0.001±0.004	0.014±0.001
21	Volume of grey matter in Inferior Frontal Gyrus pars opercularis left	-0.001±0.004	0.014±0.001
22	Volume of grey matter in I IV Cerebellum left	-0.001±0.004	0.014±0.001
23	Volume of grey matter in Thalamus left	-0.001±0.004	0.014±0.001
24	Volume of grey matter in Frontal Pole right	-0.001±0.004	0.014±0.001
25	Volume of grey matter in Precentral Gyrus left	-0.002±0.004	0.013±0.001
26	Volume of grey matter in Inferior Frontal Gyrus pars opercularis right	-0.001±0.004	0.013±0.001
27	Volume of grey matter in Hippocampus right	-0.001±0.004	0.013±0.001
28	Volume of grey matter in Central Opercular Cortex right	-0.001±0.004	0.013±0.001
29	Volume of grey matter in Paracingulate Gyrus right	-0.001±0.004	0.013±0.001
30	Volume of grey matter in Temporal Fusiform Cortex posterior division right	-0.001±0.004	0.013±0.001
31	Volume of grey matter in Frontal Operculum Cortex left	-0.001±0.004	0.013±0.001
32	Volume of grey matter in Superior Temporal Gyrus anterior division right	-0.001±0.004	0.013±0.001
33	Volume of grey matter in Inferior Temporal Gyrus posterior division left	-0.001±0.004	0.013±0.001
34	Volume of grey matter in Parahippocampal Gyrus posterior division left	-0.001±0.004	0.013±0.001
35	Volume of grey matter in Heschl s Gyrus includes H1 and H2 left	-0.001±0.004	0.013±0.001
36	Volume of grey matter in Inferior Temporal Gyrus anterior division left	-0.001±0.004	0.013±0.001
37	Volume of grey matter in Superior Temporal Gyrus posterior division left	-0.001±0.004	0.012±0.001
38	Volume of grey matter in Lingual Gyrus left	-0.001±0.004	0.012±0.001
39	Volume of grey matter in Frontal Medial Cortex left	-0.001±0.004	0.012±0.001
40	Volume of grey matter in Middle Temporal Gyrus posterior division right	-0.001±0.004	0.012±0.001
41	Volume of grey matter in Inferior Frontal Gyrus pars triangularis left	-0.001±0.003	0.012±0.001
42	Volume of grey matter in Angular Gyrus right	-0.001±0.003	0.012±0.001
43	Volume of grey matter in Temporal Pole left	-0.0±0.003	0.012±0.001
44	Volume of grey matter in Frontal Pole left	-0.0±0.003	0.012±0.001
45	Volume of grey matter in Inferior Temporal Gyrus anterior division right	-0.0±0.004	0.012±0.001
46	Volume of grey matter in Planum Temporale right	0.0±0.004	0.012±0.001
47	Volume of grey matter in X Cerebellum vermis	0.0±0.004	0.012±0.001
48	Volume of grey matter in Cuneal Cortex left	0.0±0.004	0.012±0.001
49	Volume of grey matter in Middle Temporal Gyrus posterior division left	0.0±0.004	0.012±0.001
50	Volume of grey matter in I IV Cerebellum right	0.0±0.004	0.012±0.001
51	Volume of grey matter in Angular Gyrus left	0.0±0.004	0.011±0.001
52	Volume of grey matter in Inferior Temporal Gyrus posterior division right	0.0±0.004	0.011±0.001
53	Volume of grey matter in Temporal Occipital Fusiform Cortex right	0.0±0.003	0.011±0.001
54	Volume of grey matter in Crus I Cerebellum vermis	0.0±0.004	0.011±0.001
55	Volume of grey matter in Cingulate Gyrus posterior division right	0.0±0.003	0.011±0.001
56	Volume of grey matter in Juxtapositional Lobule Cortex left	0.0±0.003	0.011±0.001
57	Volume of grey matter in Middle Temporal Gyrus temporooccipital part right	0.0±0.003	0.011±0.001
58	Volume of grey matter in Subcallosal Cortex right	0.001±0.003	0.011±0.001
59	Volume of grey matter in VIIb Cerebellum vermis	0.001±0.003	0.011±0.001
60	Volume of grey matter in Precentral Gyrus right	0.001±0.003	0.011±0.001
61	Volume of grey matter in Lingual Gyrus right	0.001±0.003	0.011±0.001
62	Volume of grey matter in Parahippocampal Gyrus posterior division right	0.001±0.003	0.011±0.001
63	Volume of grey matter in Insular Cortex right	0.001±0.003	0.011±0.001
64	Volume of grey matter in Frontal Operculum Cortex right	0.001±0.003	0.011±0.001
65	Volume of grey matter in Central Opercular Cortex left	0.001±0.003	0.011±0.001
66	Volume of grey matter in Superior Frontal Gyrus right	0.001±0.003	0.011±0.001
67	Volume of grey matter in Heschl s Gyrus includes H1 and H2 right	0.001±0.003	0.011±0.001
68	Volume of grey matter in Thalamus right	0.001±0.003	0.01±0.001

69	Volume of grey matter in Superior Parietal Lobule right	0.001±0.003	0.01±0.001
70	Volume of grey matter in Cingulate Gyrus anterior division right	0.001±0.003	0.01±0.001
71	Volume of grey matter in VI Cerebellum right	0.001±0.003	0.01±0.001
72	Volume of grey matter in Pallidum right	0.001±0.003	0.01±0.001
73	Volume of grey matter in Postcentral Gyrus left	0.001±0.004	0.01±0.001
74	Volume of grey matter in Insular Cortex left	0.001±0.004	0.01±0.001
75	Volume of grey matter in Parietal Operculum Cortex left	0.001±0.004	0.01±0.001
76	Volume of grey matter in VI Cerebellum left	0.001±0.004	0.01±0.001
77	Volume of grey matter in Pallidum left	0.001±0.004	0.01±0.001
78	Volume of grey matter in Supracalcarine Cortex right	0.001±0.004	0.01±0.001
79	Volume of grey matter in Occipital Fusiform Gyrus right	0.001±0.004	0.01±0.001
80	Volume of grey matter in Superior Parietal Lobule left	0.002±0.004	0.01±0.001
81	Volume of grey matter in Temporal Pole right	0.002±0.004	0.01±0.001
82	Volume of grey matter in X Cerebellum right	0.002±0.004	0.01±0.001
83	Volume of grey matter in Brain Stem	0.002±0.004	0.01±0.001
84	Volume of grey matter in Inferior Temporal Gyrus temporooccipital part left	0.002±0.004	0.009±0.001
85	Volume of grey matter in Temporal Occipital Fusiform Cortex left	0.002±0.004	0.009±0.001
86	Volume of grey matter in Amygdala right	0.002±0.004	0.009±0.001
87	Volume of grey matter in Middle Frontal Gyrus left	0.002±0.004	0.009±0.001
88	Volume of grey matter in Postcentral Gyrus right	0.002±0.004	0.009±0.001
89	Volume of grey matter in Frontal Orbital Cortex left	0.002±0.003	0.009±0.001
90	Volume of grey matter in Amygdala left	0.002±0.004	0.009±0.001
91	Volume of grey matter in V Cerebellum left	0.002±0.004	0.009±0.001
92	Volume of grey matter in Middle Frontal Gyrus right	0.002±0.004	0.008±0.001
93	Volume of grey matter in Superior Frontal Gyrus left	0.002±0.004	0.008±0.001
94	Volume of grey matter in Superior Temporal Gyrus posterior division right	0.002±0.004	0.008±0.001
95	Volume of grey matter in Parahippocampal Gyrus anterior division right	0.002±0.004	0.008±0.001
96	Volume of grey matter in Supramarginal Gyrus posterior division left	0.002±0.004	0.008±0.001
**97**	**Volume of grey matter in Frontal Medial Cortex right**	**0.002±0.004**	**0.008±0.001**
98	Volume of grey matter in Frontal Orbital Cortex right	0.002±0.004	0.008±0.001
99	Volume of grey matter in IX Cerebellum vermis	0.002±0.004	0.007±0.001
100	Volume of grey matter in Paracingulate Gyrus left	0.002±0.004	0.007±0.001
101	Volume of grey matter in VIIIb Cerebellum vermis	0.002±0.003	0.007±0.001
102	Volume of grey matter in V Cerebellum right	0.002±0.003	0.007±0.001
103	Volume of grey matter in Lateral Occipital Cortex superior division right	0.002±0.003	0.007±0.001
104	Volume of grey matter in Parahippocampal Gyrus anterior division left	0.002±0.003	0.007±0.001
105	Volume of grey matter in Middle Temporal Gyrus temporooccipital part left	0.002±0.003	0.007±0.001
106	Volume of grey matter in Crus II Cerebellum vermis	0.002±0.003	0.006±0.001
107	Volume of grey matter in Supracalcarine Cortex left	0.002±0.003	0.006±0.001
108	Volume of grey matter in Temporal Fusiform Cortex anterior division left	0.002±0.003	0.006±0.001
109	Volume of grey matter in Parietal Operculum Cortex right	0.002±0.003	0.006±0.001
110	Volume of grey matter in Precuneous Cortex right	0.002±0.003	0.006±0.001
111	Volume of grey matter in IX Cerebellum left	0.002±0.003	0.006±0.001
112	Volume of grey matter in Temporal Fusiform Cortex anterior division right	0.002±0.003	0.006±0.001
113	Volume of grey matter in X Cerebellum left	0.002±0.003	0.005±0.001
114	Volume of grey matter in VI Cerebellum vermis	0.002±0.003	0.005±0.001
115	Volume of grey matter in Ventral Striatum left	0.002±0.003	0.005±0.001
116	Volume of grey matter in VIIIa Cerebellum vermis	0.002±0.003	0.005±0.001
117	Volume of grey matter in Cuneal Cortex right	0.002±0.003	0.005±0.001
118	Volume of grey matter in Lateral Occipital Cortex superior division left	0.002±0.003	0.005±0.001
119	Volume of grey matter in VIIIb Cerebellum left	0.002±0.003	0.005±0.001
120	Volume of grey matter in Precuneous Cortex left	0.002±0.003	0.005±0.001
121	Volume of grey matter in Putamen left	0.002±0.003	0.005±0.001
122	Volume of grey matter in Occipital Fusiform Gyrus left	0.002±0.003	0.005±0.001
123	Volume of grey matter in Ventral Striatum right	0.002±0.003	0.004±0.001
124	Volume of grey matter in IX Cerebellum right	0.002±0.002	0.004±0.001
125	Volume of grey matter in Occipital Pole left	0.002±0.002	0.004±0.001
126	Volume of grey matter in VIIIb Cerebellum right	0.002±0.002	0.004±0.001
127	Volume of grey matter in Intracalcarine Cortex right	0.002±0.002	0.004±0.001
128	Volume of grey matter in VIIIa Cerebellum left	0.002±0.002	0.004±0.001
129	Volume of grey matter in Putamen right	0.002±0.002	0.004±0.001
130	Volume of grey matter in Crus I Cerebellum left	0.002±0.002	0.003±0.0
131	Volume of grey matter in Crus I Cerebellum right	0.002±0.001	0.003±0.0
132	Volume of grey matter in Occipital Pole right	0.002±0.001	0.003±0.0
133	Volume of grey matter in Intracalcarine Cortex left	0.002±0.001	0.003±0.0
134	Volume of grey matter in VIIIa Cerebellum right	0.001±0.001	0.002±0.0
135	Volume of grey matter in VIIb Cerebellum right	0.001±0.001	0.001±0.0
136	Volume of grey matter in VIIb Cerebellum left	0.001±0.001	0.001±0.0
137	Volume of grey matter in Crus II Cerebellum left	0.001±0.001	0.001±0.0

Table A12 List of most frequent words in the literature related to the neural correlates of DT extracted following the univariate analysis pipeline. The frequency of occurrence of each word was calculated across all papers for each individual brain region, and normalized between 0 and 1, with 1 corresponding to the most frequent word. The frequency scores of the 5 words with the highest values were added for all the brain regions related to DT. The neural correlates of DT were extracted following the univariate analysis pipeline.

Word	Cumulative frequency of occurrence
visual	3.646944
stimulus	1.530769
lesion	1.298545
learning	1.282701
age	1.174014
schizophrenia	1.046582
reward	1.000000
motor	1.000000
asd	1.000000
auditory	1.000000
speech	0.980220
word	0.893082
speed	0.806647
motion	0.800604
reversal	0.800448
number	0.754717
layer	0.716012
choice	0.665919
early	0.557692
field	0.556604
social	0.430085
pattern	0.419872
measure	0.407051

Table A13 List of most frequent words in the literature related to the neural correlates of DT extracted following the multivariate analysis pipeline. The frequency of occurrence of each word was calculated across all papers for each individual brain region, and normalized between 0 and 1, with 1 corresponding to the most frequent word. The frequency scores of the 5 words with the highest values were added for all the brain regions related to DT. The neural correlates of DT were extracted following the multivariate analysis pipeline.

Word	Cumulative frequency of occurrence
visual	2.572749
age	2.000000
motor	1.800718
lesion	1.315089
stimulus	1.273666
object	1.000000
reward	1.000000
dyslexia	0.978723
learning	0.901345
schizophrenia	0.872340
fmri	0.851064
speed	0.806647
motion	0.800604
reversal	0.800448
state	0.797872
impairment	0.728905
ad	0.728905
measure	0.718133
layer	0.716012
anisotropy	0.697761
sensory	0.679458
injury	0.679104
brainstem	0.670429
choice	0.665919
approach	0.660448
clinical	0.604966
stimulation	0.519187
shape	0.299484
representation	0.237522

Table A14 List of most frequent words in the literature related to the neural correlates of AS extracted following the multivariate analysis pipeline. The frequency of occurrence of each word was calculated across all papers for each individual brain region, and normalized between 0 and 1, with 1 corresponding to the most frequent word. The frequency scores of the 5 words with the highest values were added for all the brain regions related to AS. The neural correlates of AS were extracted following the multivariate analysis pipeline.

Word	Cumulative frequency of occurrence
memory	3.755957
age	3.240682
auditory	2.000000
motor	1.721180
schizophrenia	1.408927
asd	1.000000
reward	1.000000
error	1.000000
anxiety	1.000000
speech	0.980220
ad	0.960422
word	0.893082
behavior	0.811451
visual	0.779874
number	0.754717
dopamine	0.734452
emotional	0.704225
emotion	0.686620
sensory	0.679458
size	0.678261
development	0.653623
surface	0.638070
splenium	0.633333
relationship	0.620053
clinical	0.604966
hemisphere	0.604348
lower	0.603217
shell	0.589339
conflict	0.561404
stimulus	0.530769
stimulation	0.519187
case	0.512415
expression	0.504573
action	0.501385
microstructural	0.493404
subject	0.470330
drug	0.454097
trial	0.435826
pattern	0.429840
synaptic	0.419207
monitoring	0.419206
learning	0.380335
stress	0.297256
primary	0.293073
hearing	0.273535
memory	3.755957

Table A15 List of most frequent words in the literature related to the neural correlates of AS extracted following the multivariate analysis pipeline. The frequency of occurrence of each word was calculated across all papers for each individual brain region, and normalized between 0 and 1, with 1 corresponding to the most frequent word. The frequency scores of the 5 words with the highest values were added for all the brain regions related to AS. The neural correlates of AS were extracted following the multivariate analysis pipeline.

Word	Cumulative frequency of occurrence
memory	2.000000
social	1.679775
behavior	1.595449
emotional	1.332494
learning	1.281681
fear	1.221097
schizophrenia	1.099290
auditory	1.000000
semantic	1.000000
anxiety	1.000000
reward	1.000000
speech	0.980220
priming	0.849558
pain	0.840708
reversal	0.800448
automatic	0.752212
age	0.733766
impairment	0.727273
development	0.727273
damage	0.707792
choice	0.665919
lesion	0.609865
stimulus	0.530769
expression	0.504573
crf	0.472081
subject	0.470330
synaptic	0.419207
stress	0.297256

Supplementary Results

Feature selection using random forest and comparison between AS and AB models

The feature selection step was repeated using Random Forest (RF). The same train/test split applied in case of the linear regression analysis was used. The four datasets (Volume, FA, Thickness and Intensity) were combined and input into a RF algorithm to identify to top 15 relevant features across all feature sets. Fifteen features were selected in order to be consistent with the number of features that were identified in the original pipeline. To achieve this, a RF model was trained on the training set and the features were ranked based on their Gini importance score. The top 15 features were extracted and used to train a RF model which was then tested on the held-out test set. This process was repeated twice to predict AS and AB. The performance of the AS and AB models was compared using the Mean Squared Error (MSE) and the R2 on the held-out test set.

The R2 on the test set was 0.015 for AS and 0.011 for AB, while the MSE was comparable between the two models (0.0163 for AB and 0.0166 for AS), with the difference being negligible.

The identified neural correlates (i.e., top 15 features) of AS were: mean thickness of the trasversetemporal hemisphere (left), mean intensity of CC Mid anterior whole brain, and vessel (left), volume of grey matter in amygdala (left and right), Brain Stem, Crus II Cerebellum (left and right), Frontal Pole (right), Hippocampus (left), Pars opercularis (right), Pallidum (left), Postcentral Gryus (left), and Temporal Fusiform cortex (left).
